# Primary Thyroid Tuberculosis Masquerading as a Follicular Neoplasm With Tracheal Compression: A Case Report

**DOI:** 10.7759/cureus.100321

**Published:** 2025-12-29

**Authors:** João Varanda, Daniel Martins, Hugo Pais Moreira, Antónia Póvoa, Bela Pereira

**Affiliations:** 1 General Surgery, Unidade Local de Saúde de Gaia/Espinho, Porto, PRT; 2 Surgery, Centro Hospitalar Vila Nova de Gaia e Espinho, Gaia, PRT

**Keywords:** follicular adenoma, granulomatous thyroiditis, multinodular goiter, pcr diagnosis, thyroid tuberculosis

## Abstract

Thyroid tuberculosis (TB) is a rare form of extrapulmonary infection that can mimic thyroid neoplasms. Diagnosis is challenging due to nonspecific imaging and cytology. We report a case of a 77-year-old female with a history of subtotal gastrectomy for gastric adenocarcinoma. Surveillance CT revealed right-lobe-predominant thyroid enlargement causing tracheal compression. Ultrasound showed a multinodular goitre with a 4.1-cm solid nodule (Thyroid Imaging Reporting and Data System (TI-RADS) 3) and a 1.7-cm hypoechoic nodule (TI-RADS 4). Fine-needle aspiration cytology (FNAC) of the smaller nodule suggested a follicular neoplasm. The right thyroid lobectomy was performed. Histology revealed a follicular adenoma with epithelioid granulomas, and polymerase chain reaction (PCR) confirmed *Mycobacterium tuberculosis (M. tuberculosis)* complex DNA. The patient completed standard anti-tuberculosis therapy and remained asymptomatic at eight months of follow-up. TB should be considered in patients with nodular thyroid disease, particularly when granulomatous inflammation or compressive symptoms are present. Molecular testing facilitates accurate diagnosis, guides therapy, and helps avoid unnecessary surgery.

## Introduction

Extrapulmonary tuberculosis (EPTB) accounts for 15-20% of all tuberculosis cases worldwide, frequently affecting lymph nodes, pleura, bones, and genitourinary organs [1]. Thyroid involvement is extremely rare (<1%) even in tuberculosis-endemic regions [2-4], likely due to the gland’s high vascularity, iodine-rich colloid, and encapsulation, which provide resistance to mycobacterial infection [5-8].

Clinical presentations are variable, ranging from solitary nodules and multinodular goiters to diffuse enlargement or abscesses, sometimes causing compressive symptoms such as dysphagia, dyspnea, or stridor [9-13]. These features overlap with thyroid malignancy or benign nodular disease, making early diagnosis challenging. Due to similarities with malignancy, patients may undergo unnecessary surgery for an infection treatable with medication. Imaging with ultrasound or a CT scan is essential but nonspecific. Nodules may appear solid, cystic, or mixed, occasionally with calcifications [14,15]. Fine-needle aspiration cytology (FNAC) may be inconclusive due to sparse granulomas or the absence of acid-fast bacilli [16-19].

## Case presentation

**Figure 1:**
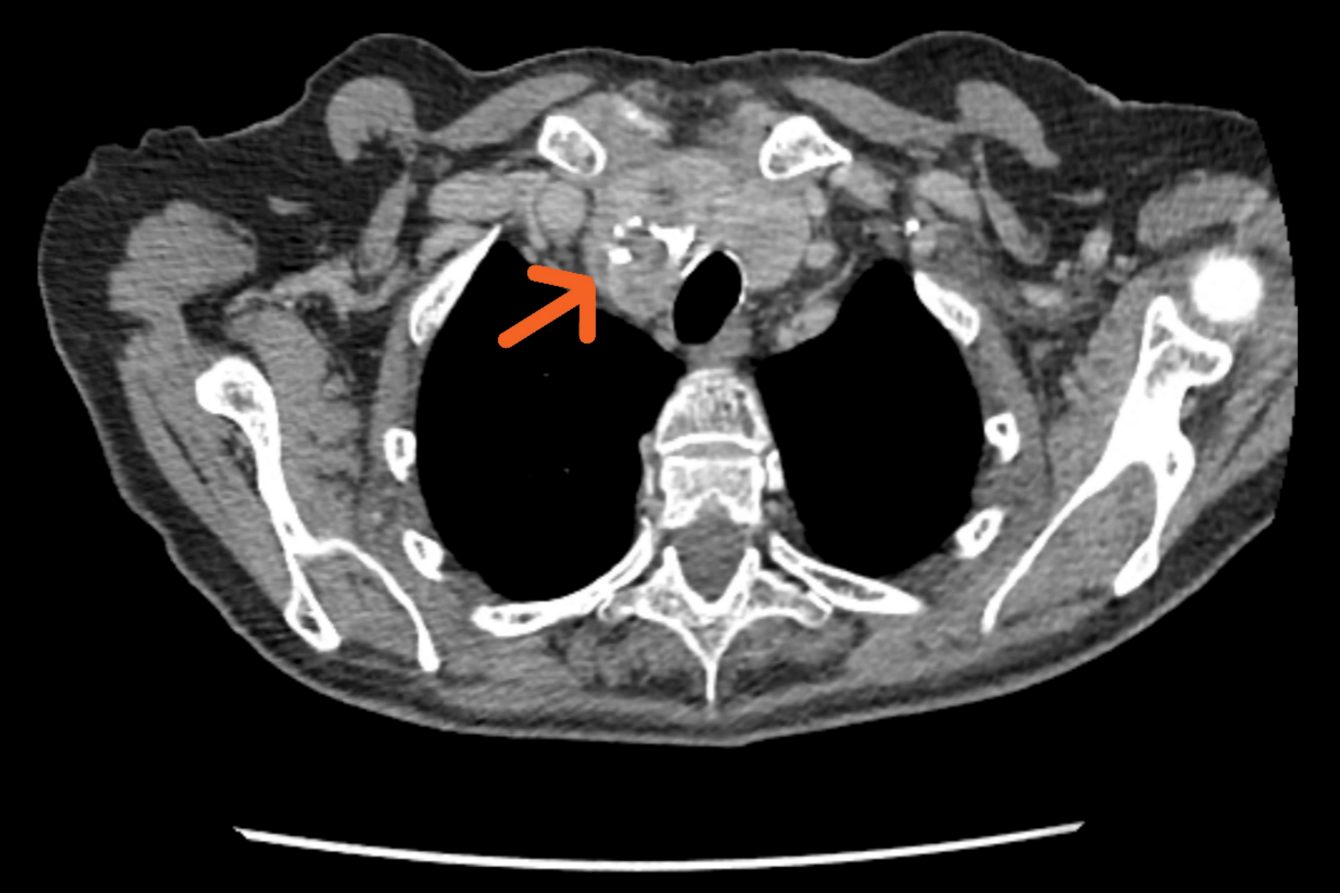
Axial CT scan of the neck CT: computed tomography

A 77-year-old female with prior subtotal gastrectomy for gastric adenocarcinoma, hypertension, and dyslipidemia (on valsartan and simvastatin) underwent routine surveillance. CT imaging revealed right-lobe-predominant thyroid enlargement causing tracheal compression and minor deviation (Figure 1). Thyroid function was normal.

**Figure 2:**
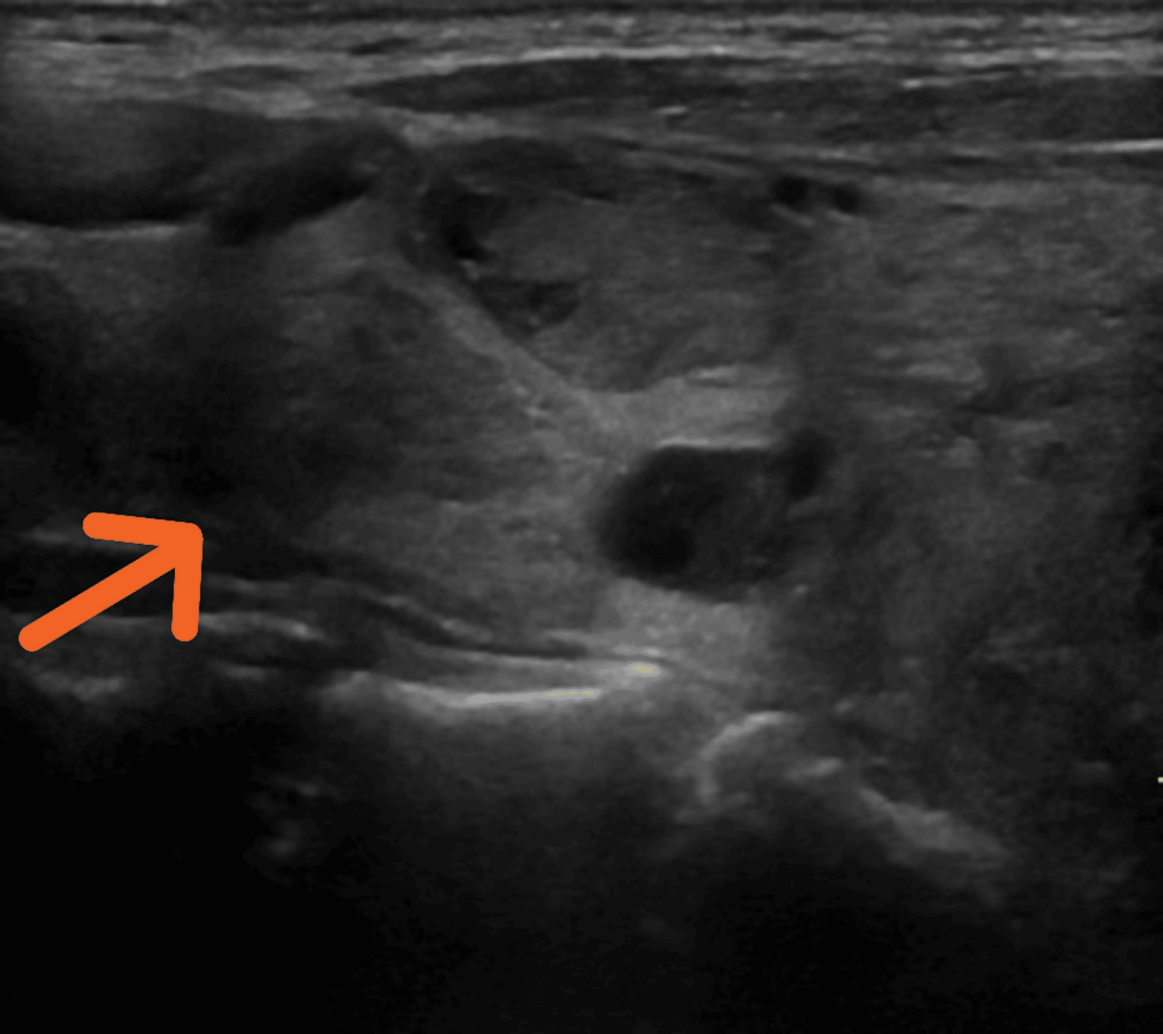
Nodules of the right thyroid lobe

Neck ultrasound revealed a multinodular goiter: right lobe: 4.1-cm mixed, predominantly solid nodule with macrocalcifications (Thyroid Imaging Reporting and Data System (TI-RADS) 3) - right lobe: 1.7-cm predominantly solid, mildly hypoechoic nodule (TI-RADS 4) (Figure 2) - multiple smaller nodules in the left lobe. No cervical lymphadenopathy was observed. While the larger nodule caused compression, the smaller one was biopsied due to higher suspicion features.

FNAC of the 1.7-cm nodule suggested a follicular neoplasm (Bethesda IV). A right thyroid lobectomy was performed due to compressive symptoms and indeterminate cytology. Postoperative recovery was uneventful, apart from a minor seroma clinically detected and managed conservatively.

**Figure 3:**
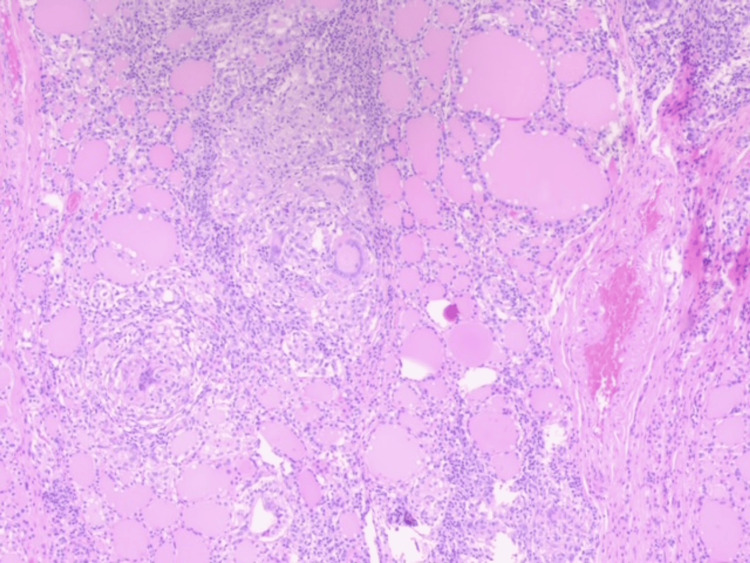
Thyroid tissue with well-defined epithelioid granulomas

**Figure 4:**
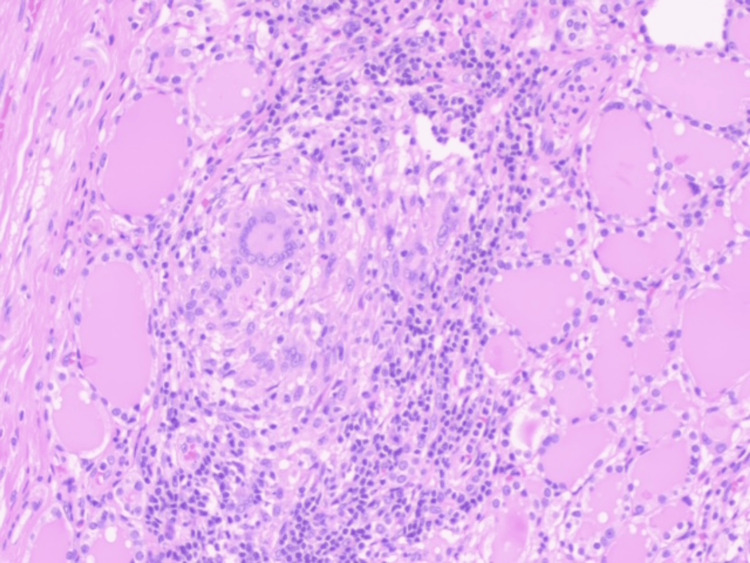
Epithelioid granuloma with giant multinucleic cells and inflamatory lymphocytic infiltration

Histopathology revealed a follicular adenoma with epithelioid granulomas (Figures 3, 4). Ziehl-Neelsen staining was negative. Polymerase chain reaction (PCR) testing of FFPE (formalin-fixed, paraffin-embedded tissue) confirmed *Mycobacterium tuberculosis (M. tuberculosis)* complex DNA. The patient completed standard anti-tuberculosis therapy and remained asymptomatic with no recurrence at the eight-month follow-up.

## Discussion

Thyroid TB is rare, accounting for <1% of thyroid surgical specimens [2-4,7]. Presentations are diverse, including nodules, multinodular goitre, abscess, or diffuse enlargement [14-16]. Compressive symptoms are uncommon but clinically significant, as in this case, in which a right-lobe-dominant nodule caused tracheal deviation. Ultrasound is essential for assessing nodule characteristics and mass effect, but cannot distinguish TB from malignancy [17,18]. FNAC may be limited by sparse granulomas and often negative acid-fast staining [19]. PCR enables definitive diagnosis from FNAC or surgical specimens, even in paucibacillary tissue. Previous reports support the utility of PCR in confirming TB when cytology is indeterminate [4,5].

Medical therapy with standard anti-TB regimens remains the first-line treatment [1,3,6]. Surgical intervention is reserved for cases of diagnostic uncertainty, compressive symptoms, or abscess drainage [7,15]. In this case, TB was not suspected preoperatively, and lobectomy was performed due to cytological suspicion and possible airway compromise. Histopathological examination combined with PCR confirmed TB, thereby preventing unnecessary extensive surgery.

Cases of thyroid TB causing compressive symptoms are rare. Fewer than 300 cases of thyroid TB have been reported in the global medical literature to date [20], making this condition extremely rare even in TB-endemic regions. Several reviews have emphasized this rarity: a 2006 review reported approximately 200 cases worldwide up to that time; a 2017 systematic review identified only seven cases reported in Western European countries since 2010; and a 2006 literature review found 76 cases that met its selection criteria. Teferi et al. reported a giant thyroid abscess causing airway obstruction [9], while Pachipala et al. described nodules larger than 3 cm managed surgically in combination with anti-TB therapy [4]. Many reports highlight the challenge of distinguishing TB from carcinoma, particularly with hypoechoic, solid, or calcified nodules.

The clinical lessons from this case are as follows: a high index of suspicion for TB should be maintained in patients with nodular thyroid disease accompanied by granulomatous inflammation or compressive symptoms, even among elderly or post-oncologic populations. PCR should be used to obtain a rapid and definitive diagnosis when cytology and conventional histology are inconclusive. Surgical intervention should be individualized for cases with diagnostic uncertainty or significant mass effect, while acknowledging that medical therapy remains the cornerstone of treatment.

## Conclusions

This report describes thyroid TB presenting as a follicular lesion with tracheal compression, highlighting diagnostic challenges, molecular confirmation, and clinical management. Although rare, thyroid TB is an important differential diagnosis in nodular thyroid disease, as it can mimic follicular neoplasms and cause compressive symptoms, leading to potential misdiagnosis when relying on cytology and imaging alone. PCR testing on FNAC or surgical specimens enables definitive diagnosis and timely anti-TB therapy, though its role in preventing unnecessary surgery should be interpreted cautiously in this case, as PCR was performed postoperatively. Clinicians, particularly in TB-endemic regions, should maintain a high index of suspicion for this entity to facilitate prompt recognition and multidisciplinary management. Reporting such rare cases contributes to improved understanding of clinical presentations, diagnostic approaches, and management strategies, while broader recommendations regarding routine molecular testing or future research should be framed cautiously.
